# Pancreas and islet morphology in cystic fibrosis: clues to the etiology of cystic fibrosis-related diabetes

**DOI:** 10.3389/fendo.2023.1269139

**Published:** 2023-11-23

**Authors:** Sarah S. Malik, Diksha Padmanabhan, Rebecca L. Hull-Meichle

**Affiliations:** ^1^ Department of Pharmacology, University of Washington, Seattle, WA, United States; ^2^ Research Service, Veterans Affairs Puget Sound Health Care System, Seattle, WA, United States; ^3^ Seattle Institute for Biomedical and Clinical Research, Seattle, WA, United States; ^4^ Division of Metabolism, Endocrinology, and Nutrition, Department of Medicine, University of Washington, Seattle, WA, United States

**Keywords:** cystic fibrosis, cystic fibrosis-related diabetes, exocrine pancreas, islet, duct, beta cell, microenvironment

## Abstract

Cystic fibrosis (CF) is a multi-organ disease caused by loss-of-function mutations in *CFTR* (which encodes the CF transmembrane conductance regulator ion channel). Cystic fibrosis related diabetes (CFRD) occurs in 40-50% of adults with CF and is associated with significantly increased morbidity and mortality. CFRD arises from insufficient insulin release from β cells in the pancreatic islet, but the mechanisms underlying the loss of β cell function remain understudied. Widespread pathological changes in the CF pancreas provide clues to these mechanisms. The exocrine pancreas is the epicenter of pancreas pathology in CF, with ductal pathology being the initiating event. Loss of CFTR function results in ductal plugging and subsequent obliteration. This in turn leads to destruction of acinar cells, fibrosis and fatty replacement. Despite this adverse environment, islets remain relatively well preserved. However, islet composition and arrangement are abnormal, including a modest decrease in β cells and an increase in α, δ and γ cell abundance. The small amount of available data suggest that substantial loss of pancreatic/islet microvasculature, autonomic nerve fibers and intra-islet macrophages occur. Conversely, T-cell infiltration is increased and, in CFRD, islet amyloid deposition is a frequent occurrence. Together, these pathological changes clearly demonstrate that CF is a disease of the pancreas/islet microenvironment. Any or all of these changes are likely to have a dramatic effect on the β cell, which relies on positive signals from all of these neighboring cell types for its normal function and survival. A thorough characterization of the CF pancreas microenvironment is needed to develop better therapies to treat, and ultimately prevent CFRD.

## 1 Introduction

**Figure 1:**
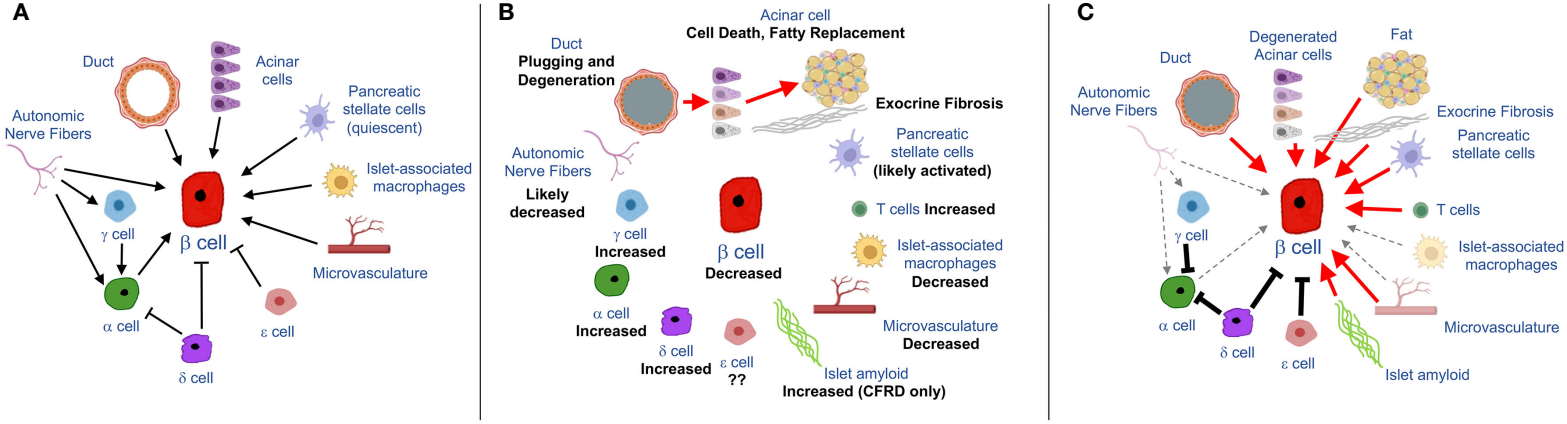
Schematics showing input from cells of the pancreas/islet to the β cell under normal **(A)** and CF **(B, C)** conditions. **(A)** Summary of physiological/homeostatic input to β cell from exocrine-, endocrine or islet-associated cell types. **(B)** Pathological features of the exocrine pancreas in CF, emanating from ductal pathology, as indicated by red arrows, along with changes in abundance of islet endocrine cells and support cells (nerve, vascular and immune cells). **(C)** hypothesized implications for β cell function and/or survival based on CF pancreatic pathology. Red arrows denote detrimental (chiefly inflammatory) signals derived from diseased cell types. Dashed grey arrows denote decreased input to the β cell due to loss of nerve fibers, macrophages or vasculature. Solid black lines denote increased inhibitory input to β and α cells (due to increased abundance of δ, γ and potentially also ϵ cells) some elements in the figure were made using BioRender.

Cystic fibrosis (CF) is the most prevalent life-limiting autosomal recessive disease affecting populations of Northern European descent (1), although it is important to recognize that CF affects all racial/ethnic groups (2, 3). Mutations in the cystic fibrosis transmembrane conductance regulator (CFTR) leads to multi-organ disease, with progressive lung disease representing the leading cause of premature death (1, 4). With substantial advances in therapies, life expectancy of people with CF in the United States has progressively increased, and is now ~45 years (3). With this increase in survival, however, other features of CF have emerged, including CF-related diabetes (CFRD). CFRD affects 20% of adolescents and ~40-50% of adults with CF (3, 5) and, importantly, is an important risk factor for worsened CF lung disease. CFRD results in a 4-6 fold greater mortality rate compared to people with CF who do not have diabetes (6–9). The inadequate release of insulin from the islet β cell underlies the development of CFRD (10–14), manifest as a characteristic blunted and delayed insulin response to nutrient stimulation. One potential contributor is an impairment in the incretin axis (i.e. action of the hormones glucagon like peptide 1 and gastric inhibitory polypeptide to enhance insulin release in response to glucose) (15, 16). However, much remains unknown about the etiology of insulin deficiency in CF, limiting the development of improved treatments for CFRD. The focus of this article is the islet/pancreas microenvironment whose constituent cell types, under normal conditions, provide critical signals to maintain β cell function, identity and survival (**Figure 1A**). In CF, profound and widespread changes occur in this microenvironment (**Figure 1B**), providing clues to novel mechanisms that may underlie loss of insulin release in this disease (**Figure 1C**).

## 2 Pathology and dysfunction of the exocrine and endocrine pancreas in CF

### 2.1 Exocrine pancreas pathology and dysfunction in CF

Within the pancreas, CFTR is predominantly expressed, at very high levels, in pancreatic ductal epithelial cells (PDECs) (17). There, its regulation of Cl^-^ and HCO_3_^-^ transport is critical for maintaining luminal pH and water balance and maintaining acinar-derived digestive enzymes in a dilute, inactive form (18, 19). Loss of CFTR function results in increased protein concentration and subsequent plugging of the ductal lumen (18). This results in duct obliteration, which in turn leads to destruction of acinar cells and fatty replacement of the exocrine pancreas. This pathology is strikingly similar to that seen in other diseases of the exocrine pancreas, including chronic pancreatitis (20). Fibrosis is also a common feature of CF pancreas disease. Its etiology remains understudied, but likely involves activation of pancreatic stellate cells (PSCs), as has been demonstrated in other exocrine pancreas diseases (21). Under normal conditions, PSCs contribute to normal tissue structure and homeostasis (21). In disease, however, activation of PSCs results in their proliferation and trans-differentiation to myofibroblasts, leading to synthesis and deposition of a fibrotic, proinflammatory extracellular matrix (21, 22). A role for PSCs in CF pancreas fibrosis is therefore likely, but remains to be clarified.

In CF, exocrine pancreas pathology is initiated very early in life [even *in utero* (23)], and while pancreatic morphology appears relatively preserved in infants (<1 year of age), substantial abnormalities are seen by 5 years of age (24). Consequently, exocrine pancreas insufficiency occurs in the vast majority (~85%) of people living with CF (18). Moreover, in the minority that retain exocrine pancreatic function, there is still evidence of damage to their pancreas (18) with reports of pancreatitis occurring in individuals upon treatment with highly effective CFTR modulator therapies (HEMT) (25). Therefore, exocrine pancreas pathology is itself a major, debilitating feature of the CF disease process. Moreover, as described below in section 3, emerging evidence suggests it may also be a key contributor to the pathogenesis of CFRD.

### 2.2 Endocrine pancreas pathology and dysfunction

Given the extensive disruption of the exocrine pancreas in CF, it is surprising that islets remain relatively intact. However, the number and/or arrangement of endocrine cell types that make up the islet are significantly altered in CF.

#### 2.2.1 Islet β cells

The islet β cell is the most abundant islet endocrine cell and is the source of the hormone insulin, which is required for maintenance of glucose homeostasis. Impaired insulin release is common among people living with CF (10, 11, 13, 26), and is the key contributing factor to the onset of CFRD (as it is for all forms of diabetes). Defects in processing of insulin from its precursor, proinsulin have been described in CF models (27), a further indication of β cell dysfunction.

In human autopsy pancreas specimens, decreases in β cell area in adult CF subjects without diabetes when compared to non-CF controls have been reported in some (28–31) but not all studies (24, 32); data from these studies are summarized in **Table 1**. β cell loss does not appear to differ based on the major form of exocrine pathology (i.e. fatty vs. fibrotic) (31) although it appears that a greater degree of β cell loss is observed in younger CF subjects (24). Similarly, most studies report a modest but not severe loss of β cell mass in CFRD (24, 32–34).

**Table 1:** Summary of findings from morphometric studies examining islet endocrine cell types in donors with CF (no diabetes) and/or CFRD.

Study	Ref #	Donor information		Comparison: CF vs non-CF					Comparison: CFRD vs CF no diabetes				
		Donor samples/group	Donor age range	Beta cell		Alpha cell	Delta cell	PP cell	Beta cell		Alpha cell	Delta cell	PP cell
				per islet	per pancreas				per islet	per pancreas			
Iannucci et al., 1984	(33)	4-6	Adolescent-adult	–	–	–	–	–	Decreased	–	Increased	Increased	No change
Abdul-Karim et al., 1986	(28)	4-11	Adolescent-adult	Decreased	–	No change	Increased	–	Decreased	–	No change	Increased	–
Soejima et al., 1986	(34)	6-34	Pediatric-adult	–	–	–	–	–	Decreased	–	No change	Increased	–
Lohr et al., 1989	(31)	6-23	Adolescent-adult	Decreased	–	Increased	Increased	Increased	–	–	–	–	–
Couce et al., 1996	(29)	12-16	Adolescent-adult	Decreased	–	–	–	–	No change	–	–	–	–
Bogdani et al, 2017	(24)	3-8	Adult	Decreased	No change	Increased	Increased	No change	Decreased	No change	No change	No change	No change
Hart et al, 2018	(30)	5-7	Adult	No change	Decreased	Trend	Increased	–	–	–	–	–	–
Hull et al., 2018	(32)	17-20	Pediatric-adult	No change	No change	Increased	Trend	Increased	Trend	No change	Increased	No change	No change
Bogdani et al, 2017	(24)	11-16	Young (<4 years)	Decreased	Decreased	Increased	Increased	Increased	–	–	–	–	–

"-" denoted that this measurement was not determined in that study.

The mechanisms underlying loss of β cells in CF are not well understood. β cell apoptosis may be a contributor, as has been described in one study (24). However, in T2D, it has been noted that the observed decrease in β cell mass is much greater than can be accounted for by the rate of β cell apoptosis (35). This, and other work, led to the concept that loss of β cell identity and not solely β cell death may contribute to the decreased number of functional β cells that are seen in diabetic conditions (35, 36). In β cells, markers of differentiated identity include the transcription factors, MAFA, NKX6.1, PDX1, PAX6, NKX2-2, ISL1, NEUROD1, FOXO1 and FOXA2 (37) as well as key components of the β cell secretory pathway (including GCK, SLC2A2, SLC2A1 and INS itself). In CF, islet cell de-differentiation has been suggested based on work identifying islet cells that are positive for chromogranin A but negative for islet hormones, and the presence of polyhormonal cells (38). However, which cell type(s) may lose identity markers remains unclear. Only one study to date has investigated β cell identity markers in CF. Bulk RNAseq in islets from CF vs. non-CF donors revealed no significant changes in expression of key β cell transcription factors (*MAFA, NKX6.1, PDX1, PAX6, ISL1*) or components of the β cell secretory pathway (*INS, SLC2A2, SLC2A1, GCK, ABCC8, GLP1R*) (30).

This suggests that both β cell mass and identity remain largely unperturbed in CF, despite the extensive destruction of the surrounding exocrine pancreas, and that strategies to improve their function could be a viable means to treat and even prevent CFRD.

#### 2.2.2 Islet α cells

The islet α cell is the second most abundant islet endocrine cell type, whose main function is the secretion of glucagon, a hormone which has a range of metabolic effects, including stimulation of hepatic glucose production (39, 40). In contrast to the decrease seen in β cell abundance, α cells have been shown to be increased in autopsy pancreas specimens from donors with CF both with and without diabetes, using immunohistochemical and electron microscopy approaches (24, 30–32). However, despite the increased abundance of α cells, glucagon secretion (like insulin secretion) is impaired in CF, in response to a range of stimuli including arginine and hypoglycemia (10, 41).

A possible contributor to α cell dysfunction in CF is loss of cell identity. In islets from CF donors, a significant decrease in *ARX* expression (30), one of the transcription factors critical for α cell identity (37), was observed. While more research is needed, an imbalance between the abundance vs. function/identity of α cells seems to be a key feature of islet pathology in CF.

#### 2.2.3 Islet δ, γ and ϵ cells

The population of islet δ cells is variable and much less abundant than α or β cells. δ cells secrete somatostatin and act as intra-islet paracrine negative regulators of α and β cells (42). Plasma levels of somatostatin have been reported to be increased in CF (43). However, the half-life of islet-derived somatostatin is very short and as such peripheral levels are likely not reflective of those within the islet. Therefore, the status of δ cell function in CF remains unknown. Morphologically, similar to α cells, the abundance of δ cells is significantly increased or shows an upward trend in CF islets based on multiple studies (24, 28, 30–32, 34), suggesting that local concentrations of somatostatin in the CF islet may be elevated.

γ cells make up only about 5% of islet area and are found more commonly in the head of the pancreas. These cells release pancreatic polypeptide (PP), and PP levels have been shown to be indicative of vagal input to the pancreas. In general, the abundance of γ cells, like α and δ cells, is increased in CF (24, 32), although this has not been a universal finding (33), and γ cells do not seem to be consistently increased in islets from CF donors with and without diabetes (32). Despite some inconsistencies in reports of the abundance of γ cells, there is a profound defect in PP release in CF, revealing another example of a disconnect between the abundance and function of islet endocrine cells in this disease.

ϵ cells are the fifth and final type of endocrine islet cell type in the pancreas. ϵ cells produce ghrelin, which is principally known as a gut hormone that activates the growth hormone secretagogue receptor (44). Circulating levels of ghrelin (specifically the active, acylated form) are increased in CF (45). However, a more accurate picture of islet ϵ cell function in CF required direct examination of ghrelin levels within the pancreatic islet; this has not been determined to date.

Regarding cell identity markers, less is known about the nature of these in δ, ϵ, and γ cells, although there is overlap between transcription factors expressed in ϵ and γ cells, with similarity to those expressed in α cells (37). Furthermore, HHEX has been shown to contribute to δ cell differentiation (46). Whether these identity markers are compromised in the CF islet and could thereby contribute to impaired function of δ, γ and ϵ cells remains to be determined.

### 2.3 Pathological changes in non-endocrine islet cells

#### 2.3.1 Microvasculature

The pancreatic islet has an extensive microvascular capillary network which acts as a conduit for transportation of nutrients to islet endocrine cells and delivery of islet hormones to peripheral tissues (47–50). In addition, the islet microvasculature has emerged as a key source of supportive signals (e.g. growth factors, extracellular matrix components) that are necessary to maintain islet β-cell function and survival. Both of the main constituent cell types, islet endothelial cells which form the capillary wall and the constrictive pericytes which surround them, have been shown to play this positive role in modulating β cell function and survival (48, 49, 51–57).

In both T1D and T2D structural defects in the islet vasculature have been observed, including thickening and fragmentation of capillaries and an apparent increase in vascular density (58, 59). Moreover, in models of T2D, endothelial cells attain an inflamed, pro-adhesive phenotype (49, 55, 60, 61) that renders them incapable of supporting insulin release (55). In CF, changes in islet microvascular density appear to differ profoundly from what is seen in T1D and T2D. Specifically, preliminary data from our lab demonstrate a substantial decrease in capillary density in islets and exocrine pancreas (62). Moreover, islet bulk RNAseq data from human donors with CF exhibit increases in inflammatory markers, including key indicators of endothelial inflammation/activation including *SELE* and *IL6* (30). Together, these data suggest that while there is a decrease in islet endothelial cell abundance within CF islets, those remaining endothelial cells are highly inflamed.

While some data exist regarding islet endothelial cells in CF, the other main constituent of the islet microvasculature, the pericyte, remains entirely unstudied. Pericytes surround microvascular endothelial cells and are responsible for control of blood flow (63, 64) but are also critical for microvessel stability and the prevention of vessel leakage and inflammation. Loss of pericyte attachment and/or cell death has been defined as a critical pathological event in multiple vascular diseases including tumor growth, diabetic retinopathy and kidney fibrosis, and the close interaction between endothelial cells and pericytes has been shown to be disrupted in islets in T2D and models thereof (61, 63, 65). These data suggest that loss of microvascular stability could be a common pathological feature of the diabetic islet. Whether the same is true in CF/CFRD remains an important unanswered question.

#### 2.3.2 Innervation

It has long been appreciated that pancreatic islets are innervated by sympathetic, parasympathetic and sensory neurons (66, 67). Parasympathetic input stimulates, while sympathetic input inhibits insulin release (66, 67). Moreover, sympathetic input to the α cell is required for the glucagon response to hypoglycemia (39). Islet innervation in CF has not been well-studied, although a decrease in PGP9.5+ nerve fiber density has been reported in the CF pig pancreas (68). The profound defect in PP release in people living with CF is suggestive of a loss of vagal input to the pancreas, and the loss of islet (and exocrine) capillary density would also be expected to be reflective of impaired innervation to both pancreas compartments, given the known close association of the vasculature and autonomic nerve fibers (67). However, more data are clearly needed to delineate the state of pancreatic innervation in human CF.

#### 2.3.3 Immune cells

While (increased) macrophages within the islet have been reported to have detrimental effects under diabetic conditions (69), it is now well established that resident intra-islet macrophages are critical for β-cell growth, regeneration following injury and function/glucose homeostasis (70–75). We previously reported that increased IL-1β positivity is a common and early feature of the islet in CF (32). Based on data from T2D, whereby increased IL-1β production is indicative of an increase in the number and/or activation state of macrophages, this observation prompted an investigation of the abundance of islet macrophages in CF. Surprisingly, we and others found an almost complete absence of intra-islet macrophages in adolescents and adults with CF (24, 32) (regardless of diabetes status), despite the continued presence of macrophages in exocrine pancreas.

Islets also contain other resident leukocyte populations, including relatively rare T-lymphocytes. Increased T cell infiltration is a hallmark of autoimmune T1D and has also been described in islets in CF pancreas sections taken from children and adults (24, 30). Flow cytometry analysis of CF donor islets leukocyte populations revealed a relatively abundant CD3+ T cell population, which largely (~60%) consisted of CD8+ T cells (30).

#### 2.3.4 Islet amyloid

Amyloids are aggregates of misfolded proteins and have been linked to the development of numerous diseases. In the islet, amyloid deposits contain as their unique component islet amyloid polypeptide (IAPP), which is a normal secretory product of the β cell (76). Islet amyloid deposition has been recognized as a feature of islets in CF for several decades (29) and, unlike many of the other features described above is pathognomonic of CFRD, occurring in 60-70% of cases compared to only 0-20% of individuals with CF without diabetes (24, 29, 32). Moreover, islet amyloid does not appear to be a pathological feature in children with CF (24), although, deposition in CFRD appears to be markedly accelerated compared to the classically-described islet amyloid seen in subjects with T2D, where amyloid is associated with established disease in individuals around the 6^th^-7^th^ decade of life (76, 77).

## 3 Potential mechanisms underlying CFRD

### 3.1 Investigation of intrinsic effects of CFTR in the β cell

Perhaps the most straightforward explanation for β cell dysfunction in CF would be that mutated CFTR has an intrinsic effect to impair insulin secretion in β cells. Indeed, some studies provide evidence for β-cell CFTR expression/activity and an effect on insulin release (27, 78–80). Conversely, studies using RNAseq, *in situ* hybridization and immunohistochemistry show that β cell expression of CFTR is very low and/or occurs in a small proportion of β cells (30, 81–85). Moreover, patch-clamp electrophysiology failed to detect a forskolin-activated chloride currents in human β cells (which would be consistent with CFTR activity) (30), and CFTR modulators were unable to alter insulin release in isolated human islets in response to glucose and cAMP-mediated activation (30). These latter data are consistent with no impact on insulin release or glucose tolerance in mouse models with β cell deletion of *Cftr* (30). Therefore, while intrinsic effects of CFTR in β cells may occur, it seems likely that extrinsic effects of the profoundly altered microenvironment in the CF pancreas are also important in dysregulation of insulin release.

### 3.2 Impact of pancreatic ductal pathology on β cell function

There is clear support from the literature for a role of exocrine pancreas pathology/dysfunction on insulin release, best illustrated by the fact that CF patients with pancreatic insufficiency have a greater deficit in insulin and glucagon release vs. those with residual exocrine pancreatic function (10, 13, 14). As mentioned, exocrine pathology is initiated in young children, although it remains relatively normal until around 1 year of age, suggesting there may be a window of opportunity for prevention. Therefore, understanding mechanisms whereby early stages of exocrine pancreas pathology may exert detrimental effects on the β cell is important and timely.

Some evidence exists to suggest that ductal pathology may contribute directly to β-cell dysfunction. First, small intercalating ducts are located in close proximity to islets, making them appropriately positioned to exert effects on β cells (86–88). Second, the limited available data examining an effect of pancreatic ductal epithelial cells (PDECs) on β cell function under normal circumstances generally show a positive effect. Specifically, co-incubation of human islets with PDECs ameliorated the decline in β-cell function which is observed with long term culture (89, 90). It has also been reported that co-transplantation of islet with PDECs improved islet transplantation outcomes (91–93) In contrast, one study showed that PDEC-secreted factors resulted in increased basal insulin release (with no effect on GSIS), and worsened islet transplantation outcomes (94). The reason for the discrepant data is not clear, but the authors of the latter study suggest that “inflammation may mediate the deleterious effects of ductal cells on islet cells”.

Several studies suggest that loss of CFTR expression/activity from PDECs can impair insulin release (82, 85, 95). Knockdown of CFTR in isolated islets (from ferrets or human donors) was sufficient to impair insulin release (82, 85). This interpretation is based on the authors’ demonstration that CFTR expression in isolated islets was restricted to ductal epithelial cells [the presence of ductal cells within isolated islet preparations is well supported in the literature (82, 96–99)]. A separate study developed a model whereby islets were cultured in close proximity to human PDECs (95). Here, acute CFTR inhibition in those PDECs resulted in decreased insulin release from islets. While this study had the advantage of a PDEC-selective intervention, it had some limitations, such as use of the pharmacological inhibitor CFTR (inh)-172, which has been shown to have non-specific effects (82, 100–102).

Further, PDECs with defective CFTR function likely release proinflammatory mediators that could impair insulin release from β cells. Human pluripotent stem cell (hPSC)-derived PDECs from CF donors express a proinflammatory transcriptome (103). Moreover, PDECs from CF ferrets release a pro-inflammatory secretome (104). Several of the differentially regulated proteins identified in the latter study have been reported to impact pathways known to affect β cell/glucose metabolism (105–109). For example, IGFBP7 was found to be downregulated in PDECs, although it was increased in pancreatic stellate cells (PSCs) from CFTR^-/-^ ferrets, and exogenous treatment of wild-type whole islets from ferrets, IGFBP7 resulted in altered insulin secretion (104). Together, these data strongly suggest that PDEC dysfunction, especially in the early stages of CF pancreas disease may contribute to impaired β cell function/survival.

### 3.3 Impact of acinar pathology on β cell function

It is well-established that pancreatic ductal pathology is also the key initiator of pancreatic acinar destruction in CF (18, 23, 24, 110), and in turn the profound destruction and remodeling of acinar cells is a likely source of signals that may negatively impact the β cell.

There is evidence in the literature to support the existence of an acinar-islet axis: insulin is known to modulate pancreatic acinar cell function (111, 112), while pancreatic acinar-derived products affect islet cell function and proliferation (113–116). This suggests that normal acinar function is important in maintaining β cell function and, conversely, that acinar pathology or loss in CF could contribute to impaired insulin release.

Potential mediators of negative effects of inflamed acinar cells on the islet come from *in vitro* studies of chronic pancreatitis (which also manifests acinar dysfunction/destruction) (117). These studies show increased production of proinflammatory cytokines such as TNF-α and IL-1β from pancreatic acinar cells, which are known to mediate impaired insulin release and/or β cell death. Recent work focused on MODY8 (which occurs due to mutations in the acinar lipase *CEL*) showed that defective acinar cells were able to induce ER stress and β cell secretory dysfunction (118). Additionally, the extensive fibrosis and infiltrating fat that characterize the CF pancreas may themselves release factors that could impact β cell survival and function, although the nature of such factors remain unknown.

### 3.4 Endocrine cell cross-talk

Disruptions to the islet microenvironment likely also contribute to the insulin deficiency that characterizes CF. In α cells, several studies suggest that CFTR may have intrinsic effects (119, 120), although whether CFTR is expressed/active in α cells remains controversial and understudied. It is now well recognized that paracrine effects of α cell products (predominantly glucagon and GLP-1) are required for optimal β cell function. However, the increased α cell abundance in the face of impaired insulin release in CF suggests an impairment in that paracrine axis. Indeed, new data suggests that gene expression related to cell identity and hormone secretion is altered in α cells from CF donors (30, 121), and that β cell GLP-1R expression is also decreased (121), both of which would be consistent with impaired α-to-β cell communication within the CF islet.

For other islet endocrine cells, fewer data exist. However, the increased abundance of δ cells suggests that local islet somatostatin levels may be elevated, which would be expected to suppress both insulin and glucagon release. For PP cells, these may also be increased in CF, despite a suppression of circulating PP levels, suggesting again that local islet levels may be increased. Given that PP has been shown to inhibit glucagon secretion through the PPYR-1 receptors in α cells (122), an increase in islet PP could be another means by which glucagon (and thereby insulin) release could be impaired in CF. For ϵ cells, as mentioned, the status of islet ghrelin production in CF is unknown. However, circulating levels are increased (45) which, given the known effect of ghrelin to suppress insulin release (but enhance glucagon release) could also impact islet function. Taken together, dysregulation of islet endocrine cell composition and/or function likely play a critical role in perturbing insulin release in CF.

### 3.5 Changes in the pancreas/islet microenvironment

It is highly likely that the decrease in islet vascular density reported in our preliminary study has a profound effect on insulin secretion into the peripheral circulation. In fact, the profile of insulin release classically seen in CF, namely a blunted and delayed insulin response (10, 13, 14), could be explained almost entirely by the impaired delivery of nutrients to the islet along with inefficient delivery of insulin to the peripheral circulation that would be expected to occur in the face of decreased islet vascularization. The concept that impaired insulin release may be at least partly dependent on this loss of vascularity, also fits with data showing that β cell mass (24, 28–32) and identity (30) are relatively preserved in CF, suggesting again that “indirect” mechanisms likely explain the deficient insulin release. And, moreover, this is also consistent with data showing that when isolated islets from CF donors are studied *ex vivo* (i.e. under conditions where they are no longer dependent on vascularization for nutrient delivery and hormone release), insulin release is relatively intact (30). Of course, additional data will be required to fully test this hypothesis.

Similarly, the anticipated loss in autonomic innervation to the CF pancreas is also likely to have a major impact on islet function. This decreased innervation is likely to be a generalized effect, affecting sympathetic, parasympathetic and sensory input, as it is anticipated to occur secondary to exocrine pancreas destruction (and loss of vasculature). Therefore, CF islets may be essentially denervated, which would certainly be consistent with known defects such as impaired insulin release (e.g. due to reduced cholinergic tone), loss of the glucagon response to hypoglycemia (due to loss of sympathetic input) and essentially absent PP secretion (due to loss of vagal input).

While the activation of PSCs during fibrosis has been most widely studied in the context of pancreatic cancer and pancreatitis (21, 22) there is also some evidence to suggest the activated PSCs can be detrimental to β cells. Several studies demonstrate that activated PSCs can impair β cell development (123) induce β cell death (124–126), with some (127) but not all studies (128) also suggesting a negative impact on β cell function. Analysis of secreted factors from PSCs has been published by several groups (129–134); investigation of these data may inform our understanding of potential mechanisms underlying β cell dysfunction in CF.

Several lines of evidence suggest that the islet microenvironment in CF is highly inflamed. As described above, a diverse array of proinflammatory molecules are produced by both CFTR-defective PDECs and inflamed acinar cells in the surrounding exocrine pancreas, as well as CD8+ T-cells, which are known to produce proinflammatory cytokines, and inflamed/activated endothelial cells within the islet itself. Deposition of islet amyloid, in addition to its well-known toxic effects on β cells, has proinflammatory effects on both leukocytes and endothelial cells (61, 135, 136). Moreover, the proinflammatory cytokines IL-1β and IL-6 have both been shown to be produced by human or ferret islets, respectively, in CF (32, 82); these may derive from endocrine cells themselves, although the cellular source has not been definitively determined. Finally, the loss of intra-islet macrophages would further compromise the islet’s ability to clear cell debris or pathological material such as amyloid deposits, which are known to stimulate pathways such as inflammasome and toll like receptor signaling, further exacerbating the inflammatory milieu of the CF islet. Overall, this inflammatory assault on the islet is a prime target for therapeutic intervention, with approaches ideally targeting multiple cell types and/or inflammatory mediators.

## 4 Considerations for treatment of CFRD

The ideal treatment for CFRD would be correction of *CFTR* mutations to reverse or even prevent exocrine and endocrine pancreas disease. Highly effective CFTR modulator therapies (HEMT) have had major positive benefit in treating CF lung disease and improving nutritional status/body mass index (137–140). However, only limited data are available demonstrating their impact on insulin release and glucose tolerance [reviewed in (141)].

Since exocrine and endocrine pancreatic defects begin very early in life (12, 110, 142), CFTR modulators may need to be initiated in very young children to see substantial benefit. Encouragingly, in young children (between 4 months and 5 years old), the CFTR modulator ivacaftor shows improvements in exocrine pancreas function (143–145), with small studies (including children as young as 5 years old) also showing improved insulin release (146, 147). However, it is important to recognize that many people with CF may not be able to fully benefit from these interventions (e.g. those with already-established pancreas disease or whose CFTR mutations do not have available modulator therapies). Moreover, concerningly, the improved nutritional status with the administration of HEMTs have resulted in increased prevalence of overweight and obesity in people with CF as well as the emergence of features of metabolic syndrome (148), both of which may further increase the prevalence and/or severity of CFRD.

It seems that early adoption of HEMT may be possible in some people with CF. However, even in those people, combination of HEMT with therapies aimed at reversing or compensating for aspects of the disrupted pancreas/islet microenvironment (illustrated in **Figures 1B, C**) may well be necessary to improve β cell function. These include three major areas of intervention. First, the replacement of factors that are lost or whose release is impaired in CF may be beneficial. Incretin-based therapies fit into this category and have been shown to be efficacious in small studies of people with CF (16, 149) but their broad applicability remain to be demonstrated. Other islet-specific mediators may include glucagon or factors elicited from the islet microvasculature which are required for optimal insulin release. Second, correcting aberrant restraint on the β cell due to the upregulation of paracrine inhibitors such as somatostatin or ghrelin may be effective in restoring insulin release. Finally, a key component will likely be blockade of inflammatory mediators, which are released by multiple islet/pancreatic cell types (e.g. exocrine, vascular and adaptive immune cells; see **Figure 1C**) and which are well known to impair β cell function and identity. To develop such approaches will require efforts to generate a more comprehensive picture of the underlying pathways and mediators which result in a compromised pancreatic/islet microenvironment in CF.

## Author contributions

SM: Writing – original draft, Writing – review & editing. DP: Writing – original draft, Writing – review & editing. RH-M: Conceptualization, Supervision, Writing – original draft, Writing – review & editing.
